# Editorial Responsibility

**Published:** 1857-11

**Authors:** 


### EDITORIAL AND MISCELLANEOUS.

### Editorial Responsibility.

We wish it distinctly understood that the Editors of this Journal are equally responsible for the matter contained in the Editorial Department.

We are induced to make this statement, from the fact that an exchange has recently singled out one of the Editors for a most unjustifiable personal attack.

In addition to this, we wish it as distinctly understood, that we detest controversy, and can only be drawn into it for the defence of what we conceive to be the most sacred rights.—While we have not, perhaps, that remarkable "independence and freedom from jealousies and fears," characteristic of a certain gassy "Falstaff," we yet think that we have some conception of our rights and a somewhat determined purpose to maintain them.

